# Gut microbiota link dietary fiber intake and short-chain fatty acid metabolism with eating behavior

**DOI:** 10.1038/s41398-021-01620-3

**Published:** 2021-10-01

**Authors:** Evelyn Medawar, Sven-Bastiaan Haange, Ulrike Rolle-Kampczyk, Beatrice Engelmann, Arne Dietrich, Ronja Thieleking, Charlotte Wiegank, Charlotte Fries, Annette Horstmann, Arno Villringer, Martin von Bergen, Wiebke Fenske, A. Veronica Witte

**Affiliations:** 1 Max Planck Institute for Human Cognitive and Brain Sciences, Department of Neurology, Stephanstr. 1A, 04103 Leipzig, Germany; 2 Berlin School of Mind and Brain, Humboldt-Universität zu Berlin, Berlin, Germany; 3 Charité – Universitätsmedizin Berlin, Humboldt-Universität zu Berlin, Berlin, Germany; 4 Department of Molecular Systems Biology, Helmholtz Centre for Environmental Research GmbH - UFZ, Permoserstraße 15, 04318 Leipzig, Germany; 5 Department of Visceral and Metabolic Surgery, University Hospital Leipzig, Liebigstr. 18, 04103 Leipzig, Germany; 6 Department of Endocrinology, Diabetes and Metabolism, University Hospital Bonn, Venusberg-Campus 1, 53127 Bonn, Germany; 7 Department of Psychology and Logopedics, Faculty of Medicine, University of Helsinki, Helsinki, Finland; 8 Faculty of Medicine, University of Leipzig, Leipzig, Germany; 9 Clinic for Cognitive Neurology, University Hospital Leipzig, Leipzig, Germany; 10 Institute of Biochemistry, Faculty of Life Sciences, University of Leipzig, Leipzig, Germany

**Keywords:** Physiology, Human behaviour, Biomarkers

## Abstract

The gut microbiome has been speculated to modulate feeding behavior through multiple factors, including short-chain fatty acids (SCFA). Evidence on this relationship in humans is however lacking. We aimed to explore if specific bacterial genera relate to eating behavior, diet, and SCFA in adults. Moreover, we tested whether eating-related microbiota relate to treatment success in patients after Roux-en-Y gastric bypass (RYGB). Anthropometrics, dietary fiber intake, eating behavior, 16S-rRNA-derived microbiota, and fecal and serum SCFA were correlated in young overweight adults (*n* = 27 (9 F), 21–36 years, BMI 25–31 kg/m^2^). Correlated genera were compared in RYGB (*n* = 23 (16 F), 41–70 years, BMI 25–62 kg/m^2^) and control patients (*n* = 17 (11 F), 26–69 years, BMI 25–48 kg/m^2^). In young adults, 7 bacteria genera, i.e., Alistipes, Blautia, Clostridiales cluster XVIII, Gemmiger, Roseburia, Ruminococcus, and Streptococcus, correlated with healthier eating behavior, while 5 genera, i.e., Clostridiales cluster IV and XIVb, Collinsella, Fusicatenibacter, and Parabacteroides, correlated with unhealthier eating (all | r | > 0.4, FDR-corrected *p* < 0.05). Some of these genera including Parabacteroides related to fiber intake and SCFA, and to weight status and treatment response in overweight/obese patients. In this exploratory analysis, specific bacterial genera, particularly Parabacteroides, were associated with weight status and eating behavior in two small, independent and well-characterized cross-sectional samples. These preliminary findings suggest two groups of presumably beneficial and unfavorable genera that relate to eating behavior and weight status, and indicate that dietary fiber and SCFA metabolism may modify these relationships. Larger interventional studies are needed to distinguish correlation from causation.

## Background

Gut microbes modulate brain function and behavior via immune, endocrine, neural, and humoral routes [1]. This could play a key role in neuronal feeding circuits and overeating, as dysbiosis of the microbiota composition has been documented in psychiatric eating disorders [2] and obesity [3].

However, nutrition- or body weight-related microbial changes and their functional relevance are still relatively unclear. In mice, gastric bypass-related differences in the microbiota profile, such as a higher abundance of the genera *Escherichia (*phylum *Proteobacteria)* and *Akkermansia (*phylum *Verrucomicrobia)*, induced weight loss when transferred to germ-free animals [4]. In humans, bariatric surgery similarly led to higher overall microbiota diversity and to higher abundance of the species *Escherichia coli* and in some studies to further abundance changes within the phylum *Bacteroidetes*, such as a higher post-surgery ratio of the genera *Bacteroides to Prevotella* [5] and less *Firmicutes* (phylum level) or to more *Gammaproteobacteria* (class level) [6]. The ratio of *Bacteroides to Prevotella* at baseline predicted dietary weight loss success after 24 weeks in an intervention study in 80 overweight individuals [7]. Further, a one-week dietary intervention trial in 20 individuals found that microbial composition predicted glycemic response [8].

Human-to-mouse fecal transplant experiments further underline the causal role of specific microbiota to facilitate weight loss [9], and human-to-human fecal microbiota transplantation (FMT) experiments increased insulin sensitivity according to [10]. In a recent human study, accompanied by mouse model data, an individual’s microbiota profile, extracted from fecal samples during periods of dietary weight loss, prevented weight regain when transferred back to the same individuum orally, known as autologuos FMT [11].

Mechanistic insights into how specific gut bacteria modulate human eating behavior and weight status are still limited. The gut microbiota is supposed to affect the host’s metabolism by altering energy extraction from food, and by modulating dietary or host-derived compounds that modify the metabolic pathways of the host [12]. For example, short-chain fatty acids (SCFA) are excreted by certain gut bacteria as a result of carbohydrate fermentation, and SCFA stimulate the secretion of anorexigenic hormones, such as peptide YY (peptide tyrosine tyrosine or PYY) and glucagon-like-peptide-1 (GLP-1) in the colon, which further signal to hypothalamic nuclei as one mechanism of homeostatic regulation [13]. SCFA can also cross the blood–brain barrier and act as signaling molecules in the brain to directly modulate appetite and food-decision making [1]. First interventional studies showed that intake of butyrate (one type of SCFA) or the butyrate-producing bacteria *Akkermansia spp*. exert beneficial effects on body weight depending on treatment intention in humans [14] and on brain functions in mice [15], including reduced food intake [16]. Notably, specific pre-biotic nutrients, such as dietary fibers, are known to nourish SCFA-producing bacteria in the gut, rendering diet a potent modifier of gut–brain signaling [17].

In sum, the gut microbiome may influence feeding behavior, e.g., by modulating reward and homeostatic signaling [18, 19] and by stimulating the vagal nerve [20], in particular in dysregulated biological systems, such as in food addiction [21] or eating disorders [2]. Yet, direct knowledge if specific genera are linked to eating behavior via dietary intake and SCFA in humans is lacking. Here, we asked whether gut microbial diversity and genera abundance relate to eating behavior, and to SCFA metabolites in the colon (feces) and in the periphery (blood) in a homogenous sample of young overweight adults. In addition, we tested whether the abundance of microbiota that related to eating behavior in that overweight sample correlate with weight status, eating behavior, and treatment success (i.e., achieved weight loss) in another sample, i.e., patients at two years after bariatric surgery and control overweight/obese patients.

## Methods

### Samples characteristics and data collection

We included all participants with available microbiota datasets measured at a cross-sectional timepoint from two studies. Sample 1 comprised 27 healthy young overweight adults (9 F, 21–36 years, BMI 25–31 kg/m^2^) drawn from a randomized clinical trial (Clinical Trials registration NCT03829189), where baseline data was available from ongoing data collection until January 2021. All participants were included if following a typical Western omnivorous diet and thoroughly screened for habitual dietary patterns (exclusion criteria were assessed via an interview at pre-screening included any sort of restrictive diet (incl. vegan, vegetarian, gluten-free, lactose-free, food allergies), regular excessive caffeine intake (more than 6 cups a day), regular alcohol intake (>1.25 L beer/day or equivalent) or smoking >10 cigarettes/day). Estimated nutrient intake represents a Western style omnivorous diet (10.4 ± 3.6 g/day/1000 kcal), with lower than recommended fiber intake (intake recommendations by WHO and EU nutritional agencies state >25 g or 25–35 g of dietary fiber per day are required to meet healthy intake levels). For further information on dietary and coffee intake see SI (see *Dietary Intake*).

Sample 2 comprised 23 patients two years after Roux-en-Y gastric bypass (RYGB) surgery (see below; “good responders“: *n* = 11 (7 F), 41–70 years, BMI 25–29 kg/m^2^; “bad responders”: *n* = 12 (9 F), 31–67 years, BMI 41–62 kg/m^2^), as well as age-, gender- and BMI-matched controls (overweight: *n* = 8 (5 F), 41–58 years, BMI 25–29 kg/m^2^; obese: *n* = 9 (6 F), 26–70 years, BMI 41–48 kg/m^2^), drawn from an observational study where data collection was completed (ethics proposal 027/17-ek). To compare non-surgery but BMI-matched microbial diversity with post-surgery only datapoints, body weight-matched control groups were recruited and included for microbial analysis.

All participants donated feces (see SI) for microbiota analysis (Shannon effective [22] and relative abundance of microbiota genera), underwent anthropometric measurements, and filled in questionnaires to quantify eating behavior traits. Also, data on dietary fiber intake, hunger ratings after a standardized meal, and SCFA in blood and feces were available in sample 1 (see below).

### Microbiota assessment

To assess microbiota community structure we used 16 S rRNA gene profiling of the fecal samples. Therefore, DNA was extracted and V3-V4 variable regions of the 16 S rRNA genes were amplified by PCR and a library was constructed, followed by paired-end 2x250bp Illumina sequencing. These analyses were done by GENEWIZ Germany GmbH, Leipzig. Next, the inhouse Galaxy server using a pipeline implemented with the DADA2 R package processed raw data in fastq format. For each sample, paired-end reads were joined, low-quality reads were removed, reads were corrected, chimeras removed, and Amplicon Sequence Variants (ASVs) were obtained. Taxonomy was annotated to the ASVs using the RDP database [23]. The read counts per ASV with taxonomic annotation were normalized and relative abundances of each ASV and taxa were calculated using the R scripts Rhea. Visualization of all library-indexed genera was done as in [24] by inhouse written R-tools using ggplot2.

### Eating behavior

To characterize eating behavior traits, questionnaires based on self-report were used: the Three-Factor Eating Questionnaire (TFE-Q) (German version, [25]) and the Eating Disorder Examination Questionnaire (EDE-Q) (German version, [26]) as available for sample 1 and sample 2, respectively. The TFE-Q assesses three domains of eating behavior (cognitive restraint, disinhibition, hunger), and the EDE-Q covers the subscales dietary restraint, eating concern, weight concern, and shape concern. Scoring was performed according to the respective manuals.

### Additional analyses in sample 1

From all measurements available in sample 1 in the context of the RCT (see above), we additionally considered all available hunger ratings after a standardized meal (3 out of 4 measures, 1 with missing data) and all available dietary fiber intake data (from a quantitative food frequency questionnaire, fiber in g/day and fiber per 1000 kcal). We further considered anthropometric assessments to be of interest in this study and limited those to two major health indicators, i.e., systolic blood pressure (mean of three consecutive measurements) and relative body fat (%) obtained from bioelectrical impedance analysis (see SI). Blood was obtained in fasting state (12 ± 3 h fasted) and samples were centrifuged at 3500 revolutions per minute at 7 °C for 6 min. Serum was aliquoted within 1 h of obtainment. Processed aliquots were stored at −80 °C until data analysis. For SCFA in blood and stool, analyzed according to [27], we focused on three major and most abundant SCFAs out of eight measured, i.e., acetate, butyrate, and propionate (see SI). All other measures were not considered of interest to the current analyses.

### Obesity surgery in sample 2

For sample 2, RYGB (see SI) patients were selected for microbiota analysis based on their response to the surgical treatment. Specifically, RYGB patients were identified from the database of the University of Leipzig if their surgery dated back at least 2 years and all those were further divided in percentiles according to pre-defined relative excessive weight loss (EWL) thresholds defined more conservatively than previous literature (most common <50% EWL at 18 months, according to [28]. This resulted in 23 RYGB patients good responders: sustained EWL > 70%, mean 93% ± 4 SD, range 86–98%, *n* = 12; bad responders: sustained EWL < 40%, mean 20% ± 13 SD, range 3–35%, *n* = 11). Next, obese and overweight control patients were selected from the database based on age, sex, and BMI to match those two groups of RYGB patients. Afterwards, RYGB patients only filled in a series of questionnaires, performed cognitive tests, and donated blood for another study purpose; and fecal samples of all patients were analyzed. From this dataset, we considered of interest to the current analysis the following variables: weight loss after surgery (in kg and in BMI), all available eating questionnaire data (four EDEQ scales, see above), and microbiota genera abundances based on 16 S rRNA sequencing.

### Statistical analysis

#### Correlational analysis

Relative taxa abundance (%) on the genera level was used as primary variables of interest. Non-normally distributed variables were log- or Tukey-transformed, so that skewness of <|1| was reached (for details Supplementary Fig. 1). No observations were eliminated, instead all cases with microbiota data were complete and included. For the main analysis, 20 out of 121 genera were included as they appeared in at least 80% of individuals [29] and fed into a correlation matrix with all variables of interest in sample 1 (37 variables in total, see above), i.e., Shannon index, 3 TFEQ traits, 3 hunger ratings, body fat, systolic blood pressure, dietary fiber intake (g/day and g/1000 kcal), and 3 SCFA each in feces and blood, respectively. All values were FDR-corrected and statistical significance was set to *p* < 0.05. Those genera that were significantly associated with eating behavior (TFEQ traits and/or hunger ratings, p-FDR < 0.05) were then correlated with weight status and RYGB treatment success in sample 2. Group differences across overweight, obese, good and bad RYGB responders were tested with non-parametric Kruskal–Wallis tests. Further correlations were tested with Pearson’s correlation coefficient *r* for normally distributed variables or with Spearman’s rho for non-normally distributed variables. Explorative analysis considerations were addressed according to [30] (see Additional SI).

To further investigate, if the interplay of correlated genera—rather as a holobiont than individually—is determinative of the observed relations, the relation between correlated to non-correlated genera was computed by three composite scores (1)-(3).

1 $$\begin{array}{ll}positive\;sumscore\left( \% \right) = {relative\;abundance\left(\right. {Alistipes + Blautia + Clostridium\;XVIII}} \\ \qquad\qquad\qquad\qquad\quad+ \,Gemmiger\,+\, Roseburia\, +\, Ruminococcus + Streptococcus\left)\right.\end{array}$$

2 $$\begin{array}{ll}negative\;sumscore\left(\right. \% \left)\right. = {relative\;abundance\left(\right. {Clostridium\;IV\,+\, Clostridium\;XIVb}} \\ \qquad\qquad\qquad\qquad\quad\,\,\, + \,{Collinsella \,+ \,Fusicatenibacter + Parabacteroides} \left)\right.\end{array}$$

3 $$composite\mathop {\sum }\nolimits score\left( \% \right) = \left( 1 \right) - \left( 2 \right)$$

#### Mediation analysis

Using simple mediation analysis using medmod (https://cran.r-project.org/web/packages/medmod/index.html) in RStudio version 3.6.1, we checked for statistical mediation in sample 1 for variables showing bivariate correlations in the following paths:

i. fiber —> correlated genera or sumscores —> eating behavior (TFEQ, hunger ratings)
ii. eating behavior (TFEQ, hunger ratings) —> fiber —> correlated genera or sumscores
iii. correlated genera or sumscores —> SCFA — > eating behavior (TFEQ, hunger ratings)

Significance was set to *p* < 0.05, and the main analysis for sample 1 was corrected for multiple testing using the false-detection rate (FDR)-correction. All analyses were performed in RStudio version 3.6.1.

## Results

Characteristics of sample 1 and 2 are listed below (see Tables 1–2). Data from post-RYGB patients was on average collected 4.7 ± 1.4 years after surgery. Eating behavior traits varied across both samples, and in sample 2, restrained eating and shape/weight concerns differed between those that achieved long-term excessive weight loss after bariatric surgery compared to those that did not (good vs. bad responders, all *W* > 58.5, *p* < 0.001, Table 2, Supplementary Fig. 2).

**Table 1:** Descriptives for sample 1.

	Sample 1			
*n*, sex/gender (F/M)	27 (9 F/18 M)			
	*mean*	*SD*	*minimum*	*maximum*
age (years)	28.4	4.5	21	36
education (SES index) (score from 3 to 21) (four NAs)	15.0	2.8	8.2	19.2
BMI (kg/m2)	27.7	1.7	25.0	31.2
TFEQ cognitive restraint (sumscore)	5.6	4.1	0	13
TFEQ disinhibition (sumscore)	6.0	2.1	2	11
TFEQ hunger (sumscore)	5.0	3.4	0	12
time fasted (h)	12.5	2.7	6	18
hunger 15 min postprandial (1–8 scale)	4.2	1.7	1	7
hunger 40 min postprandial (1–8 scale)	5.3	1.3	2	7
hunger 65 min postprandial (1–8 scale)	5.3	1.4	2	8
mean systolic blood pressure (mmHg)	128.0	10.9	107.0	152.7
% fat mass (female, male)	34.7 (F) 22.8 (M)	4.2 (F) 5.2 (M)	27.3 (F) 7.6 (M)	39.8 (F) 30.7 (M)
habitual fiber intake / 1000 kcal / d (g)	10.4	3.6	4.4	20.0

**Table 2:** Descriptives for sample 2.

	Overweight				Obese				Good responders				Bad responders				Group comparison
n, sex/gender (F/M)	8 (5 F/3 M)				9 (6 F/3 M)				11 (7 F/4 M)				12 (9 F/3 M)				F/t, p
	*mean*	*SD*	*minimum*	*maximum*	*mean*	*SD*	*minimum*	*maximum*	*mean*	*SD*	*minimum*	*maximum*	*mean*	*SD*	*minimum*	*maximum*	*4 groups / RYGB only*
age (years)	53	4.3	44	58	54	14.8	26	69	51.9	9.4	41	70	54.1	11	31	67	***W*** = **1.83,** ***p*** = **0.61**
BMI (kg/m2) (pre-surgery)	–	–	–	–	–	–	–	–	45.5	7.2	34.6	61	52.7	6.6	41.6	63.6	***W*** **=** **4.74,** ***p*** = **0.03**
BMI (kg/m2) (>2 years post-surgery)	27.0	1.1	25.9	29.2	45.0	2.7	41.5	47.8	26.3	0.9	25.1	27.9	47.2	6.4	41	61.9	***W*** **=** **28.6,** ***p*** < **0.001**
time post surgery (months)	–	–	–	–	–	–	–	–	51.3	15.1	25.7	74.2	55.42	18.5	25.3	76.2	***W*** = **105,** ***p*** = **0.54**
EDEQ restraint (mean score)	–	–	–	–	–	–	–	–	0.46	1.1	0	3.6	2.1	1.5	0	5	***W*** = **72.5** ***p*** = **0.003**
EDEQ eating concern (mean score)	–	–	–	–	–	–	–	–	0.26	0.3	0	0.6	0.9	0.9	0	3	***W*** **=** **84.5,** ***p*** = **0.043**
EDEQ weight concern (mean score)	–	–	–	–	–	–	–	–	0.56	1	0	3.2	3.3	1.1	1.4	5	***W*** = **60,** ***p*** < **0.001**
EDEQ shape concern (mean score)	–	–	–	–	–	–	–	–	1.1	1	0	3.3	4.2	1.1	1.5	5.5	***W*** **=** **58.5,** ***p*** < **0.001**

**Fig. 1:**
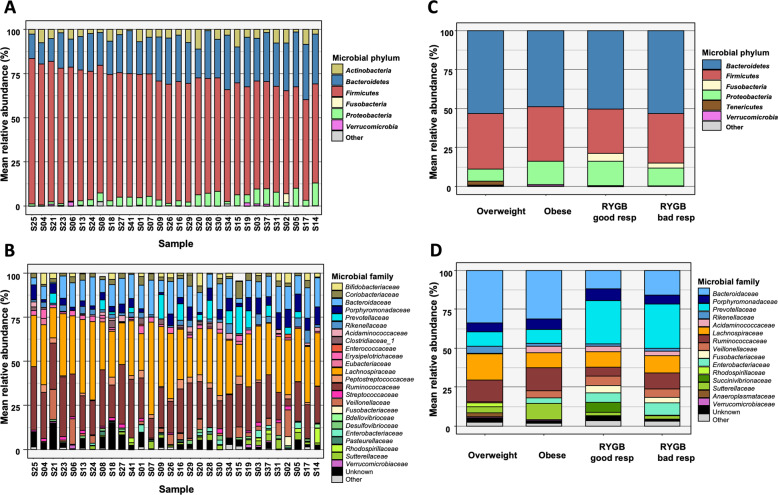
Microbiota profiling of two cross-sectional cohorts. **A** Relative abundances of phyla per subject across sample of young, overweight adults (sample 1). Sorted by Firmicutes abundance. **B** Relative abundances of family per subject across sample of young, overweight adults (sample 1). **C** Relative abundances of phyla per group for overweight and obese adults and good and bad responders after RYGB (sample 2). Colors are as in panel A. **D** Relative abundances of family per group for overweight and obese adults and good and bad responders after RYGB (sample 2). Colors are as in panel B.

Overall microbiota diversity at the phylum level was relatively comparable across participants of samples 1 and 2 except higher ratio of Firmicutes to Bacteroidetes in sample 1, and Prevotellaceae and Fusobacteriaceae families were more abundant in patients after RYGB surgery (Fig. 1, Supplementary Figs. 3–4, see SI for details). Additionally, we tested for sex/gender-specific differences in alpha diversity in sample 1 and found none (sample 1: male (*n* = 18) 111 ± 15, female (*n* = 7) 110 ± 13, *t*(13) = −0.08, *p* < 0.94). Due to limited sample size we refrained from further sex-segregated analyses, yet we encourage future meta-analyses to include our datasets (see open data).

### Microbiota, eating behavior traits, and health indicators in overweight adults

**Fig. 2:**
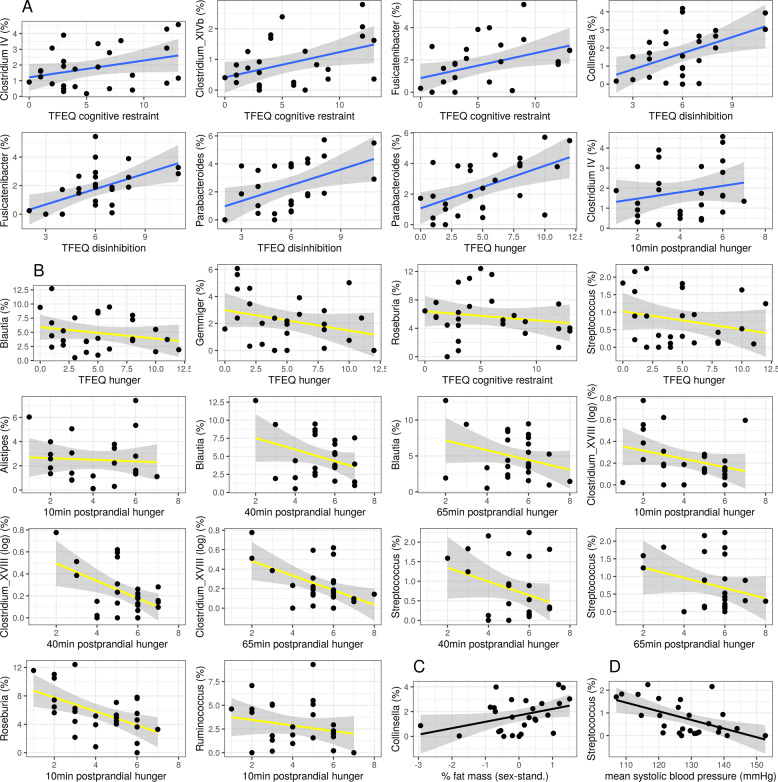
Pearson’s correlations between eating behavior traits (TFEQ and hunger ratings) or health indicators and bacterial genera in overweight adults (all | *r* | > 0.42, all p-FDR < 0.05; sample 1). **A** inversely health-related genera (blue), **B** health-related genera (yellow), **C** Collinsella and body fat mass (black), and **D** Streptococcus and mean systolic blood pressure (black).

In sample 1, effective Shannon index as a measure of alpha diversity was included into the main correlation analysis. Almost no correlation with eating behavior was significant, except that higher alpha diversity was significantly associated with 10 min-postprandial hunger (*r* = 0.59, *p* = 0.005). Further, higher relative abundance of *Collinsella* (phylum Actinobacteria), *Clostridium* IV and XIVb, *Fusicatenibacter* (all three phylum Firmicutes), and *Parabacteroides* (phylum Bacteroidetes) were related to less healthy eating behavior (higher TFEQ scores and/or higher hunger ratings, all 0.61 < |*r* | > 0.42, p-FDR < 0.05, Fig. 2A). Contrastingly, higher relative abundance of the microbial genera *Alistipes* (phylum Bacteroidetes), *Blautia*, *Clostridium* XVIII, *Gemmiger*, *Roseburia*, *Ruminococcus*, and *Streptococcus* (all phylum Firmicutes) correlated with healthier eating behavior (all 0.76 < |*r* | > 0.42, p-FDR < 0.05, Fig. 2B, Supplementary Fig. 5).

Further, *Collinsella* abundance significantly correlated with higher body fat mass (sex-standardized, *r* = 0.61, *p* < 0.001, Fig. 2C). *Streptococcus* abundance was significantly correlated with lower mean systolic blood pressure (*r* = −0.70, p-FDR < 0.001, Fig. 2D).

### Relation to dietary fiber intake and SCFA

**Fig. 3:**
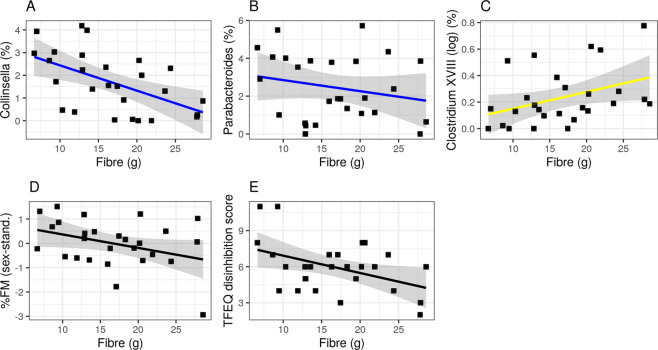
Habitual dietary fiber intake is associated with bacterial genera, body fat mass and eating traits. Pearson’s correlations shown for inversely health-related genera (blue) (**A**, **B**), health-related genera (yellow) (**C**), body fat mass (black) (**D**), and eating trait disinhibition (TFEQ) (black) (**E**) (Pearson’s correlation all 0.75 < |*r* | > 0.58, all p-FDR < 0.05; sample 1 *n* = 27).

Out of the 12 genera that were significantly associated with eating behavior (from now on called “(inversely) health-related” genera), three were associated with lower (*Collinsella* and *Parabacteroides*) or higher (*Clostridium XVIII*) dietary fiber intake (all 0.73 < |*r* | > 0.49, p-FDR < 0.05, Fig. 3A–C). Moreover, higher dietary fiber intake per se was significantly associated with lower disinhibited eating (*r* = − 0.58, p-FDR < 0.01) and lower body fat mass (*r* = −0.75, p-FDR < 0.0001, Fig. 3D–E).

SCFA concentrations in feces were highly variable and up to ~1000 times higher compared to serum for all three measured SCFA (all *t*(24) > 11.6, *p* < 0.001). Serum acetate was 2.5 times higher compared to butyrate and propionate in serum (Supplementary Table 1).

We observed that higher abundance of some of the inversely health-related genera correlated with higher levels of different SCFA in feces and serum (all *r* > 0.50, p-FDR < 0.01). In addition, most health-related genera correlated with some feces and serum SCFA markers, however revealing both positive and negative associations (only those associated with eating behavior were considered, all 0.65 < |*r* | > 0.44, p-FDR < 0.05, Supplementary Fig. 6). Note, that some genera showed differential correlations within the different SCFA, e.g., higher *Alistipes* correlated with higher acetate in both feces and serum and with fecal butyrate, but with lower fecal propionate. Moreover, considering the inversely health-related genera, *Fusicatenibacter* and *Parabacteroides* correlated significantly with higher fecal concentrations of propionate and acetate, respectively.

**Fig. 4:**
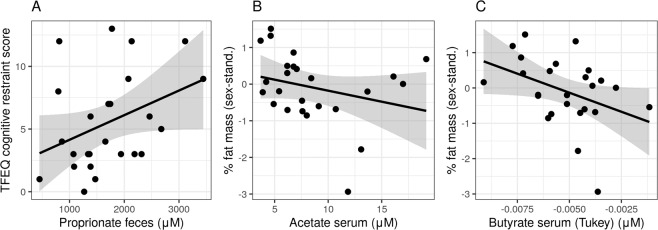
SCFA levels in feces and serum are associated with eating traits and body fat mass in overweight adults (sample 1). Pearson’s correlations shown for fecal SCFA levels and eating trait cognitive restraint (TFEQ) (r = 0.50, p-FDR = 0.014) (**A**) and serum SCFA levels with body fat mass for acetate (**B**) and butyrate (**C**) (all r > −0.43, all p-FDR < 0.04).

Also, higher fecal propionate levels correlated significantly with higher cognitive restraint eating (*r* = 0.50, p-FDR = 0.014, Fig. 4A). Higher fecal acetate, butyrate and propionate levels correlated with higher hunger ratings (all *r* > 0.45, all p-FDR < 0.04), but also serum propionate with hunger (*r* = 0.45, p-FDR = 0.03). Moreover, serum acetate and butyrate were inversely associated with body fat mass (all *r* > −0.43, all p-FDR < 0.04) (Fig. 4B–C). Notably, serum levels did not correlate with fecal SCFA concentrations (all *r* <| 0.17 | , all p-uncorr <0.86, Supplementary Fig. 7).

### Genera sumscore and mediation analyses

**Fig. 5:**
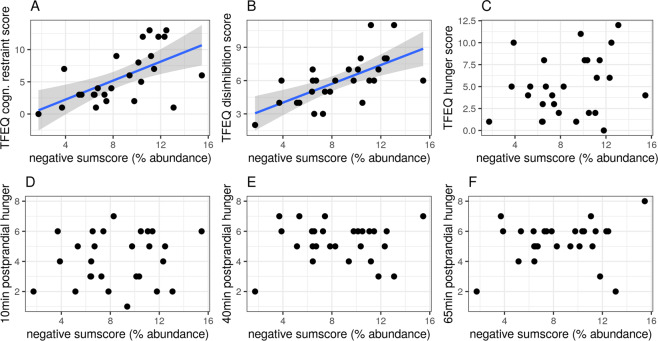
Sumscore of inversely health-related genera is positively associated with eating traits (TFEQ). Pearson’s correlations shown for microbial genera abundance sumscore for inversely health-related genera with respect to eating behavior outcomes from TFEQ and hunger ratings shown for **A**) cognitive restraint (r = 0.59, p-uncorr = 0.001) **B**) disinhibition (r = 0.65, p-uncorr < 0.001) **C**) hunger score **D**) 10 min-postprandial hunger **E**) 40 min-postprandial hunger and **F**) 65 min-postprandial hunger. Data from sample 1, all p-uncorrected.

The negative sumscore of the five inversely health-related genera abundances resulted in significant correlations for two of the eating traits (cognitive restraint *r* = 0.59, p-uncorr = 0.001; disinhibition *r* = 0.65, p-uncorr <0.001, Fig. 5A–B). The positive sumscore of the seven health-related genera abundances showed no significant associations (all p-uncorr <0.95, Supplementary Fig. 8). Neither sumscore correlated with fecal or serum SCFA levels.

Exploratory mediation path analyses of the proposed models did not show statistically significant mediating paths for differences in diet, eating behavior or hunger ratings through differences in *Parabacteroides* or positive/negative sumscores (Supplementary Tables 2–3, Supplementary Fig. 9). Considering SCFA, similar results emerged, except for acetate: here, while the direct effect c’ did not reach significance (ß = −0.3, *p* = 0.13), higher *Parabacteroides* abundance was linked with higher postprandial hunger ratings through higher fecal acetate levels (indirect effect, a*b, ß = 0.36, 95% CI [0.05 0.66], *p* = 0.02, Supplementary Table 4).

Exploratory analysis based on reviewer suggestions showed that, when adjusting the correlational analysis for body fat mass, associations with inversely health-related bacterial genera remained largely significant (TFEQ and *Clostridum XIVb*, *Collinsella*, *Fusicatenibacter*, *Parabacteroides*, all *p* < 0.05), yet positively health-related correlations do not (only for hunger ratings with *Clostridium XVIII* and *Roseburia*, all *p* < 0.05) (relating to Fig. 2, for details see SI Table 5). For fiber intake associations, only the negative association with *Collinsella* abundance and TFEQ disinhibition scores remain significant (relating to Fig. 3, for details see SI Table 5). The association between propionate levels in feces and TFEQ cognitive restraint when adjusted for body fat mass is no longer significant (relating to Fig. 4).

### Microbiota genera differences between overweight, obese, and surgery groups

**Fig. 6:**
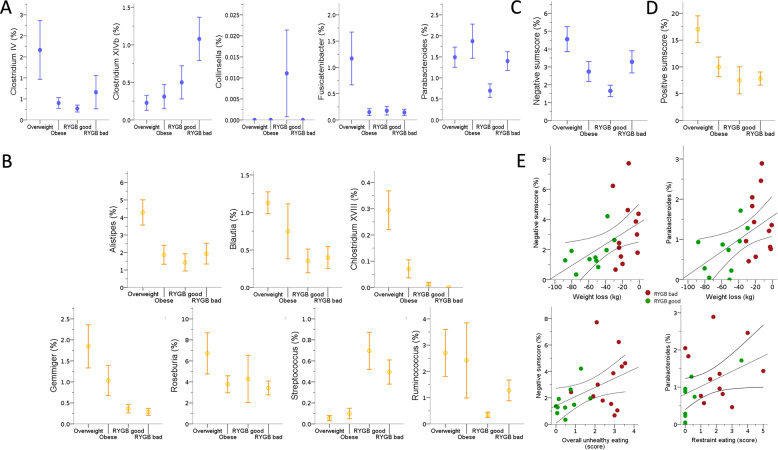
Differences in relative bacterial genera abundance across groups of overweight, obese and post-RYGB adults in sample 2. For **A** inversely health-related (blue), **B** positively related genera (yellow), **C** negative (blue), and **D** positive sumscores of all related genera (yellow) detected in sample 1. **E** Correlation of negative genera sumscore and/or Parabacteroides abundance with eating behavior and/or weight loss after RYGB surgery (green: good responders; red: bad responders). RYGB, Roux-en-Y gastric bypass. Mean + SD.

In sample 2, we aimed to confirm links between the genera of interest from sample 1 and treatment success and eating behavior. Two of the five inversely health-related genera were significantly different between groups (all H(3) >9.5, *p* < 0.023) with lower relative abundance of *Parabacteroides* in good vs. bad responders (H(1) = 4.9, *p* = 0.027) Fig. 6A). In addition, six of the seven health-related genera were more abundant in the overweight group (all H(3) >8.3, *p* < 0.036, Fig. 6B), but did not differ in the good vs. bad RYGB responders.

Considering the sumscores, we found that both sumscores differed between groups (Fig. 6C–D, all H(3)>11.3, *p* < 0.01) with the negative sumscore showing higher values in the bad vs. good RYGB responders (H(1) = 2.1, *p* = 0.036). In addition, both the positive (n.s.; H(1) = 1.9, *p* = 0.05) and the negative sumscore (H(1) = 2.02, *p* = 0.043) showed higher values in overweight vs. obese participants.

Bad vs. good RYGB responders showed higher eating restraint scores (H = 5.3, *p* = 0.022, Supplementary Fig. 1), and higher scores correlated with higher *Parabacteroides* abundance in these groups (*r* = 0.44, *p* = 0.039, Fig. 6E). Moreover, lower *Parabacteroides* abundance correlated significantly with higher weight loss after surgery (*r* = 0.49, *p* = 0.019, *n* = 20, Fig. 6E). The negative sumscore correlated with unhealthier eating behavior (mean of all EDEQ subscales, *r* = 0.47, *p* = 0.027; EDEQ restraint, *r* = 0.49, *p* = 0.022) and with less weight loss after surgery (Fig. 6E, weight, *r* = 0.53, *p* = 0.011, BMI, *r* = 0.53, *p* = 0.011).

### Potential confounders of the gut microbiome

Besides body fat mass, several confounding factors have been proposed to influence gut microbiota, such as time of day of stool collection [31], seasonality [32], coffee consumption [33], and others. Note that statistical tests showed no significant associations of the above mentioned confounders on alpha diversity in our analysis (Table 3). Influences of medication was not tested further, since medical products varied largely in sample 1 and was quite similar across patient groups in sample 2.

**Table 3:** Potential confounders on gut microbiome associations.

Potential confounder on alpha diversity	Statistical test	*p*-value
Time of day	ANOVA: grouped by AM and PM time of stool collection	*p* = 0.38
Seasonality	ANOVA: grouped by season (divided into spring, summer, autumn, winter)	*p* = 0.08
Coffee intake	ANOVA: Three groups based on mean daily coffee intake (last 7 day FFQ) 0–0.3 ml/d 0.3–45 ml/d >45 ml/d	*p* = 0.069
Sex	t.test: segregated by sex	*p* = 0.94
Medication	Not tested due to limits in sample size (esp. sample 2)	–
% body fat mass	Partial correlations for all correlations shown in Figs. 2–4 adjusted for % body fat mass	see SI Table 5

Most of these additional exploratory analyses could be performed in sample 1 only, due to limited information in sample 2.

## Discussion

Combining data from two human cross-sectional datasets, this exploratory analysis finds two groups of microbiota genera that were either positively or inversely associated with both healthier eating behavior and anthropometrics (1) in a deeply phenotyped sample of young overweight adults and (2) when comparing microbiota observed in (1) in patients showing a good or bad response two years after bariatric surgery with matched controls, respectively. More specifically, in young overweight adults, 7 bacterial genera, i.e., *Alistipes*, *Blautia*, *Clostridium XVIII*, *Gemmiger*, *Roseburia*, *Ruminococcus*, and *Streptococcus*, correlated with healthier eating behavior traits and lower subjective hunger ratings, indicating potential benefits for the host metabolism, while 5 bacterial genera, i.e., *Clostridum IV*, *Clostridium XIVb*, *Collinsella*, *Fusicatenibacter*, and *Parabacteroides*, correlated with unhealthier eating traits and higher subjective hunger ratings. *Collinsella* was further related to higher body fat mass and *Streptococcus* to lower systolic blood pressure. The health-related bacterial genera were also more abundant in the overweight good responder controls, compared to the obese bad responder controls and RYGB-operated patients, while the inversely health-related genera showed a less clear distribution across groups, with *Parabacteroides* being significantly less abundant in good vs. bad RYGB-operated patients. Moreover, relative abundance of *Parabacteroides* as well as a composite score of all inversely correlated genera, were associated with higher eating restraint and with lower post-operative weight loss across both RYGB groups. Considering diet and SCFA-related pathways, we observed that higher dietary fiber intake in overweight adults correlated with more abundant *Clostridium XVIII*, and less abundant *Collinsella* and *Parabacteroides*, as well as with healthier eating behavior and anthropometrics. While SCFA showed a rather mixed pattern of correlations with the different markers, *Fusicatenibacter* and *Parabacteroides* abundance correlated with higher fecal propionate and acetate, respectively, that again correlated with elevated hunger. Contrastingly, higher acetate and butyrate in serum correlated with lower fat mass, indicating a possible inverse association of acetate in feces and serum with respect to health indicators. Together, these results indicate that presumably beneficial and unfavorable microbiota genera relate to eating behavior and weight status, and that dietary fiber intake and SCFA metabolism may modify these relationships.

### Bacterial genera

Due to the lack of associations with alpha diversity, except for subjective hunger, it remains difficult to draw strong conclusions on relations of eating behavior and microbial diversity, measured with Shannon index, based on the present BMI-defined overweight sample. The health-related microbiota group is comprised of bacterial genera that have been described as beneficial for the host in previous literature. For example, *Alistipes* and *Blautia* were found to produce SCFA [34, 35]. Similarily, *Gemmiger*, *Roseburia*, and *Ruminococcus* belong to the families of Ruminococcaceae or Lachnospiraceae, which share a common role as active plant degraders [36]. These positive metabolic effects on the host could eventually contribute to improved adiposity control, as e.g., higher *Blautia* was correlated to lower body fat [37], *Roseburia* was linked to lower blood glucose and *Ruminococcus* to higher weight loss in mice after vertical sleeve gastrectomy via regulation of nuclear receptor binding of bile acids [38]. A microbial transfer study from human to mice showed obesity-promoting effects of the species *C. ramosum*, which is part of *Clostridium XVIII* [39]. However, studies on the genera *Clostridium XVIII* and *Streptococcus* in relation to health are scarce. Clostridia are known to be key commensals for gut homeostasis [40], but classification of the genus *Clostridium* remains challenging due to the high heterogeneity of the listed species [41]. Also, there are currently 50 species identified in the genus *Streptococcus* alone, rendering different functionality in these genera likely. Yet, we found that *Clostridium XVIII* abundance related to higher dietary fiber intake, and *Streptococcus* abundance to lower blood pressure. Indeed, fiber intake related to healthy eating behavior and lower body fat mass in overweight young adults in the present analyses may point towards rather beneficial fiber-correlating *Clostridium XVIII* and *Streptococcus* genera species that underly those associations. Moreover, these results underline the potential impact of a fiber-rich diet for health indicators. Due to the exclusive occurrence of fiber in plants, fiber-rich diets are oftentimes attributed to plant-based (vegetarian or vegan) diets, and plant-based diets have been shown extensively to be beneficial for weight status, gut, and overall health [42, 43].

Considering the inversely health-related group of microbiota, some genera were described to include pathogens, e.g., in *Clostridium XIVb* the species *C. piliforme*, the causative agent of Tyzzer’s disease [41] and *Parabacteroides* as an opportunistic pathogen in infectious diseases [44]. Of note, in the *Parabacteroides* genera, also beneficial species, e.g., *P. distasonis*, have been described [45]. The anaerobic *Collinsella* colonizes mucosal surfaces and has recently been reported to degrade potentially toxic food contaminants found in processed foods [46]. While this could be beneficial for the host, unhealthier eating behaviors (such as intake of processed food) and higher body weight could then likely be related to higher abundances of *Collinsella*. Likewise, studies showed that *Collinsella* linked to less dietary fiber intake, which is in line with our results in overweight adults, and higher weight loss in cross-sectional [47] and dietary intervention studies [48]. *Fusicatenibacter*, including the species *F. saccharivorans*, are strictly anaerobic sugar fermenters, again linking to unhealthier eating behavior and obesity [49]. The genus *Clostridum IV* however has rather been reported as beneficial SCFA producers, e.g., the species *Faecalibacterium prausnitzii* (*F. prausnitzii*), which play a noticeable role in intestinal homeostasis [50]. Yet again, those genera comprise many different species and it can also be speculated that some bacteria species or genera underlying the observed correlations could have likely been taxonomically misplaced [41]. Taken together, the negatively correlated microbiota genera seem to consist on the one hand of pathogens, indicative of a rather pro-inflammatory milieu in participants with higher weight status, which is well in line with our findings showing that higher *Parabacteroides* correlated with unhealthier eating traits and poorer weight loss maintenance in RYGB patients. On the other hand, those negatively correlated genera are comprised of those bacteria that metabolize processed food and sugars, again indicative of higher weight and unhealthy eating behavior. Future studies now need to integrate microbiota data at the species level and randomized interventional trials are required to eventually understand cause and effect of these eating behavior–microbiota–diet interrelations.

### SCFA metabolism

We could not establish reliable links between serum and fecal concentrations of those metabolites. The overall weak relationship might be explained by rapid metabolization of SCFAs, as for example butyrate is rapidly absorbed by the gut mucosa and reaches blood circulation [51], therefore, fecal levels of butyrate may not directly relate to butyrate-producing bacteria abundance nor to serum levels of butyrate. In addition, biosamples of serum and feces were not collected in a time-locked way, therefore a time difference of hours to days might have blurred potential (inverse) correlations. Indeed, it has been shown, that fecal SCFA levels decrease throughout the day due to metabolization and that overnight-fast duration influenced these results [52].

Still, we found that higher fecal SCFA levels (i.e., acetate, butyrate, and propionate) linked to higher subjective hunger ratings and also to higher cognitive restraint (i.e., propionate), whereas lower acetate and butyrate in serum correlated with higher fat mass. Statistical path analyses proposed that higher *Parabacteroides* abundance link to higher hunger through higher fecal acetate. Bearing in mind that higher fecal SCFA levels may indicate less efficient absorption in the gut, leading to lower SCFA availability in serum [53], these findings are somewhat in line with studies showing reduced appetite and less weight gain after acetate intake [1, 54]. Note however, that we did not adjust mediation statistics for multiple testing, rendering false positives likely. In addition, it has been discussed that only a minimal fraction of the colon-derived SCFA directly reaches the brain. Instead, more downstream targets of SCFA signaling might be more important for gut–brain communication, such as SCFA-induced release of GLP-1 and PYY at the gut epithelium, modulation of liver metabolism or indirect signaling via the vagus nerve [1]. Future studies could help to further disentangle the different mechanisms at play by assessing further blood-, tissue- or imaging-based biomarkers of these pathways.

In an exploratory analysis, we found that body fat mass explains some of the variance in the observed relationships, especially in those with health-related commensals, and less with inversely health-related ones. Although BMI spanned within a very homogenous overweight status group (25–30 kg/m^2^), body fat mass was quite variable (7–40%) in sample 1 and showed a significant influence of microbiome–behavior associations. This may hint to body fat as an important determinant of gut–behavior relations [55] that should receive further attention when designing dietary interventions targeting the gut microbiome.

Besides body fat, exploratory analyses showed no effects of sex/gender or common lifestyle factors on alpha diversity, yet these findings remain speculative because of small size and the cross-sectional nature of our analysis, therefore we cannot rule out that these or other factors such as medication might have confounded our analyses. Indeed, some studies reported on gut-modulating effects of nutrient supplementation such as in vitamin D [56] or vitamin B12 [57]. We recommend to document and report potential confounders in all microbiome analyses and encourage future data pooling and meta-analyses including our datasets (see open data).

### Limitations

Firstly, all analyses are based on cross-sectional data, therefore no conclusions about causal relationships can be drawn. We performed exploratory analyses centered around core hypotheses with the aim to gain more specific testable hypotheses for upcoming intervention trials. In addition, both samples are limited by size, especially with regard to the larger number of variables of interest. Due to these constraints, more elaborate statistical analyses (such as structural equation modeling) could not be performed. A major strength of this study is the inclusion of two independent samples integrating next-generation sequencing and SCFA metabolomics with psychological markers in well-characterized adults at risk for future weight gain that yielded similar associations of eating behavior with gut microbiota at the genera level.

## Conclusion

The combination of data from cross-sectional samples of overweight, obese, and post-bariatric surgery individuals showed multivariate associations between specific bacterial gut genera, particularly beneficial SCFA-producing genera and presumably unfavorable pathogens or sugar-/processed-food digesting bacteria, with anthropometrics, eating traits, and dietary fiber intake. While speculative concerning causality, our results propose key microbiota candidates for diet–gut–brain–behavior interactions in humans and may help to develop novel hypotheses how to prevent and treat unhealthy food craving through microbiotal modulation of the gut–brain axis. Longitudinal and interventional studies integrating metagenomic approaches and functional pathway analysis are needed to disentangle correlation from causality and to further characterize eating behavior-relevant microbiota genera at the species level.

## Supplementary information

Supplementary Information

Dataset Sample 1

## Data Availability

All data generated during this study are included in this published article and its supplementary information files. Raw data cannot be shared due to data protection regulations.

## References

[1] Dalile B, Van Oudenhove L, Vervliet B, Verbeke K. The role of short-chain fatty acids in microbiota–gut–brain communication. Nat Rev Gastroenterol Hepatol. 2019;16:461–478. 10.1038/s41575-019-0157-3. 10.1038/s41575-019-0157-3 31123355

[2] Seitz J, Trinh S, Herpertz-Dahlmann B (2019). The microbiome and eating disorders. Psychiatr Clin.

[3] Muscogiuri G, Cantone E, Cassarano S, Tuccinardi D, Barrea L, Savastano S (2019). Gut microbiota: a new path to treat obesity. Int J Obes Suppl.

[4] Liou AP, Paziuk M, Luevano JM Jr, Machineni S, Turnbaugh PJ, Kaplan LM. Conserved shifts in the gut microbiota due to gastric bypass reduce host weight and adiposity. Sci Transl Med. 2013;5:178ra41. 10.1126/scitranslmed.3005687. 10.1126/scitranslmed.3005687 PMC3652229 23536013

[5] Furet JP, Kong LC, Tap J, Poitou C, Basdevant A, Bouillot JL (2010). Differential adaptation of human gut microbiota to bariatric surgery-induced weight loss: links with metabolic and low-grade inflammation markers. Diabetes.

[6] Tremaroli V, Karlsson F, Werling M, Ståhlman M, Kovatcheva-Datchary P, Olbers T (2015). Roux-en-Y gastric bypass and vertical banded gastroplasty induce long-term changes on the human gut microbiome contributing to fat mass regulation. Cell Metab.

[7] Hjorth MF, Blædel T, Bendtsen LQ, Lorenzen JK, Holm JB, Kiilerich P (2019). Prevotella-to-bacteroides ratio predicts body weight and fat loss success on 24-week diets varying in macronutrient composition and dietary fiber: results from a post-hoc analysis. Int J Obes.

[8] Korem T, Zeevi D, Zmora N, Weissbrod O, Bar N, Lotan-Pompan M (2017). Bread affects clinical parameters and induces gut microbiome-associated personal glycemic responses. Cell Metab.

[9] Ridaura VK, Faith JJ, Rey FE, Cheng J, Duncan AE, Kau AL, et al. Gut microbiota from twins discordant for obesity modulate metabolism in mice. Science. 2013;341:1241214. 10.1126/science.1241214. 10.1126/science.1241214 PMC3829625 24009397

[10] Vrieze A, Van Nood E, Holleman F, Salojärvi J, Kootte RS, Bartelsman JF, et al. Transfer of intestinal microbiota from lean donors increases insulin sensitivity in individuals with metabolic syndrome. Gastroenterology. 2012;143:913–6. 10.1053/j.gastro.2012.06.031. 10.1053/j.gastro.2012.06.031 22728514

[11] Rinott E, Youngster I, Yaskolka Meir A, Tsaban G, Zelicha H, Kaplan A (2020). Effects of diet-modulated autologous fecal microbiota transplantation on weight regain. Gastroenterology.

[12] Kolodziejczyk AA, Zheng D, Elinav E (2019). Diet–microbiota interactions and personalized nutrition. Nat Rev Microbiol.

[13] Chambers ES, Morrison DJ, Frost G. Control of appetite and energy intake by SCFA: what are the potential underlying mechanisms? In: Proceedings of the Nutrition Society. Cambridge University Press, 2015, pp 328–36. 10.1017/S0029665114001657 25497601

[14] Depommier C, Everard A, Druart C, Plovier H, Van Hul M, Vieira-Silva S (2019). Supplementation with Akkermansia muciniphila in overweight and obese human volunteers: a proof-of-concept exploratory study. Nat Med.

[15] Arnoldussen IAC, Wiesmann M, Pelgrim CE, Wielemaker EM, van Duyvenvoorde W, Amaral-Santos PL (2017). Butyrate restores HFD-induced adaptations in brain function and metabolism in mid-adult obese mice. Int J Obes.

[16] Lin HV, Frassetto A, Kowalik EJ, Nawrocki AR, Lu MM, Kosinski JR (2012). Butyrate and propionate protect against diet-induced obesity and regulate gut hormones via free fatty acid receptor 3-independent mechanisms. PLoS ONE.

[17] Marques FZ, Mackay CR, Kaye DM. Beyond gut feelings: how the gut microbiota regulates blood pressure. Nat Rev Cardiol. 2017;15:20–32. 10.1038/nrcardio.2017.120. 10.1038/nrcardio.2017.120 28836619

[18] Zanchi D, Depoorter A, Egloff L, Haller S, Mählmann L, Lang UE (2017). The impact of gut hormones on the neural circuit of appetite and satiety: a systematic review. Neurosci Biobehav Rev.

[19] Alcock J, Maley CC, Aktipis CA (2014). Is eating behavior manipulated by the gastrointestinal microbiota? Evolutionary pressures and potential mechanisms. BioEssays.

[20] Goswami C, Iwasaki Y, Yada T (2018). Short-chain fatty acids suppress food intake by activating vagal afferent neurons. J Nutr Biochem.

[21] Gupta A, Osadchiy V, Mayer EA (2020). Brain–gut–microbiome interactions in obesity and food addiction. Nat Rev Gastroenterol Hepatol.

[22] Spellerberg IF, Fedor PJ (2003). A tribute to Claude Shannon (1916–2001) and a plea for more rigorous use of species richness, species diversity and the ‘Shannon–Wiener’ Index. Glob Ecol Biogeogr.

[23] Cole JR, Wang Q, Fish JA, Chai B, McGarrell DM, Sun Y, et al. Ribosomal Database Project: data and tools for high throughput rRNA analysis. Nucleic Acids Res. 2014;42:D633-42. 10.1093/nar/gkt1244. 10.1093/nar/gkt1244 PMC3965039 24288368

[24] Haange SB, Jehmlich N, Krügel U, Hintschich C, Wehrmann D, Hankir M (2020). Gastric bypass surgery in a rat model alters the community structure and functional composition of the intestinal microbiota independently of weight loss. Microbiome.

[25] Pudel V, Westenhöfer J Fragebogen zum Eßverhalten (FEV)-Handanweisung. Göttingen; Verlag für Psychologie Dr. CJ Hogrefe, 1989.

[26] Hilbert A, Tuschen-Caffier B, Karwautz A, Niederhofer H, Munsch S (2007). Eating disorder examination-questionnaire. Diagnostica.

[27] Han J, Lin K, Sequeira C, Borchers CH (2015). An isotope-labeled chemical derivatization method for the quantitation of short-chain fatty acids in human feces by liquid chromatography-tandem mass spectrometry. Anal Chim Acta.

[28] Mann JP, Jakes AD, Hayden JD, Barth JH (2015). Systematic review of definitions of failure in revisional bariatric surgery. Obes Surg.

[29] Vieira-Silva S, Falony G, Belda E, Nielsen T, Aron-Wisnewsky J, Chakaroun R (2020). Statin therapy is associated with lower prevalence of gut microbiota dysbiosis. Nature.

[30] Simmons JP, Nelson LD, Simonsohn U (2011). False-positive psychology: undisclosed flexibility in data collection and analysis allows presenting anything as significant. Psychol Sci.

[31] Liang X, FitzGerald GA (2017). Timing the microbes: the circadian rhythm of the gut microbiome. J Biol Rhythms.

[32] Koliada A, Moseiko V, Romanenko M, Piven L, Lushchak O, Kryzhanovska N (2020). Seasonal variation in gut microbiota composition: cross-sectional evidence from Ukrainian population. BMC Microbiol.

[33] González S, Salazar N, Ruiz-Saavedra S, Gómez-Martín M, de Los Reyes-Gavilán CG, Gueimonde M (2020). Long-term coffee consumption is associated with fecal microbial composition in humans. Nutrients.

[34] Parker BJ, Wearsch PA, Veloo ACM, Rodriguez-Palacios A (2020). The genus Alistipes: gut bacteria with emerging implications to inflammation, cancer, and mental health. Front Immunol.

[35] Zhang X, Zhao Y, Zhang M, Pang X, Xu J, Kang C (2012). Structural changes of gut microbiota during berberine-mediated prevention of obesity and insulin resistance in high-fat diet-fed rats. PLoS ONE.

[36] Biddle A, Stewart L, Blanchard J, Leschine S (2013). Untangling the genetic basis of fibrolytic specialization by Lachnospiraceae and Ruminococcaceae in diverse gut communities. Diversity.

[37] Ozato N, Saito S, Yamaguchi T, Katashima M, Tokuda I, Sawada K (2019). Blautia genus associated with visceral fat accumulation in adults 20–76 years of age. npj Biofilms Microbiomes.

[38] Ryan KK, Tremaroli V, Clemmensen C, Kovatcheva-Datchary P, Myronovych A, Karns R (2014). FXR is a molecular target for the effects of vertical sleeve gastrectomy. Nature.

[39] Woting A, Pfeiffer N, Loh G, Klaus S, Blaut M (2014). Clostridium ramosum promotes high-fat diet-induced obesity in gnotobiotic mouse models. MBio.

[40] Lopetuso LR, Scaldaferri F, Petito V, Gasbarrini A (2013). Commensal Clostridia: leading players in the maintenance of gut homeostasis. Gut Pathog.

[41] Yutin N, Galperin MY (2013). A genomic update on clostridial phylogeny: Gram-negative spore formers and other misplaced clostridia. Environ Microbiol.

[42] Medawar E, Huhn S, Villringer A, Veronica Witte A (2019). The effects of plant-based diets on the body and the brain: a systematic review. Transl Psychiatry.

[43] Streppel MT, Arends LR, Van’t Veer P, Grobbee DE, Geleijnse JM (2005). Dietary fiber and blood pressure: a meta-analysis of randomized placebo-controlled trials. Arch Intern Med.

[44] Boente RF, Ferreira LQ, Falcão LS, Miranda KR, Guimarães PL, Santos-Filho J (2010). Detection of resistance genes and susceptibility patterns in Bacteroides and Parabacteroides strains. Anaerobe.

[45] Wang K, Liao M, Zhou N, Bao L, Ma K, Zheng Z (2019). Parabacteroides distasonis alleviates obesity and metabolic dysfunctions via production of succinate and secondary bile acids. Cell Rep.

[46] Wolf AR, Wesener DA, Cheng J, Houston-Ludlam AN, Beller ZW, Hibberd MC (2019). Bioremediation of a common product of food processing by a human gut bacterium. Cell Host Microbe.

[47] Gomez-Arango LF, Barrett HL, Wilkinson SA, Callaway LK, McIntyre HD, Morrison M (2018). Low dietary fiber intake increases Collinsella abundance in the gut microbiota of overweight and obese pregnant women. Gut Microbes.

[48] Frost F, Storck LJ, Kacprowski T, Gärtner S, Rühlemann M, Bang C (2019). A structured weight loss program increases gut microbiota phylogenetic diversity and reduces levels of Collinsella in obese type 2 diabetics: a pilot study. PLoS ONE.

[49] Takada T, Kurakawa T, Tsuji H, Nomoto K (2013). Fusicatenibacter saccharivorans gen. nov., sp. nov., isolated from human faeces. Int J Syst Evol Microbiol.

[50] Guo P, Zhang K, Ma X, He P (2020). Clostridium species as probiotics: potentials and challenges. J Anim Sci Biotechnol.

[51] Parada Venegas D, De la Fuente MK, Landskron G, González MJ, Quera R, Dijkstra G (2019). Short chain fatty acids (SCFAs)mediated gut epithelial and immune regulation and its relevance for inflammatory bowel diseases. Front Immunol.

[52] Kaczmarek JL, Musaad SMA, Holscher HD (2017). Time of day and eating behaviors are associated with the composition and function of the human gastrointestinal microbiota. Am J Clin Nutr.

[53] Calderón-Pérez L, Gosalbes MJ, Yuste S, Valls RM, Pedret A, Llauradó E (2020). Gut metagenomic and short chain fatty acids signature in hypertension: a cross-sectional study. Sci Rep.

[54] Frost G, Sleeth ML, Sahuri-Arisoylu M, Lizarbe B, Cerdan S, Brody L (2014). The short-chain fatty acid acetate reduces appetite via a central homeostatic mechanism. Nat Commun.

[55] Le Roy CI, Bowyer R, Castillo-Fernandez JE, Pallister T, Menni C, Steves CJ, et al. Dissecting the role of the gut microbiota and diet on visceral fat mass accumulation. Sci Rep 2019;9. 10.1038/s41598-019-46193-w. 10.1038/s41598-019-46193-w PMC6611773 31278309

[56] Singh P, Rawat A, Alwakeel M, Sharif E, Al Khodor S (2020). The potential role of vitamin D supplementation as a gut microbiota modifier in healthy individuals. Sci Rep.

[57] Rowley CA, Kendall MM (2019). To B12 or not to B12: five questions on the role of cobalamin in host-microbial interactions. PLOS Pathog.

